# Rapid disease progress in a PVOD patient carrying a novel EIF_2_AK_4_ mutation: a case report

**DOI:** 10.1186/s12890-020-01186-8

**Published:** 2020-07-06

**Authors:** Xiaofang Zeng, Fan Chen, Anandharajan Rathinasabapathy, Tangzhiming Li, Agila Adnan Ali Mohammed Mohammed, Zaixin Yu

**Affiliations:** 1 Department of Cardiology, Xiangya Hospital, Central South University, 87 Xiangya Road, Changsha, 410008 Hunan China; 2 Division of Allergy, Pulmonary, and Critical Care Medicine, Vanderbilt University Medical Center, Nashville, TN USA; 3 Department of Cardiology, Shenzhen People’s Hospital, Second Clinical Medical College of Jinan University, First Affiliated Hospital of Southern University of Science and Technology, 1017 Dongmen North Road, Shenzhen, Guangdong China

**Keywords:** Pulmonary veno-occlusive disease, Pulmonary hypertension, EIF2AK4, Case report

## Abstract

**Background:**

Pulmonary veno-occlusive disease (PVOD) and pulmonary arterial hypertension (PAH) share an overlapping disease phenotype. Hence it is necessary to distinguish them.

**Case presentation:**

Our 14-year-old female patient admitted with progressive shortness of breath, dizziness, and fatigue even after minimal physical activity was clinically suspected for PAH, based on her previous history. Her chest computed tomography artery reported the presence of PVOD triad features - subpleural thickened septal lines, ground-glass nodules/opacities and mediastinal lymphadenopathy. Because of her weak physical stature, a lung biopsy was not performed; however, the genetic testing identified a novel heterozygous *EIF2AK4* mutation at c.4833_4836dup (p.Q1613Kfs*10) - the dominant susceptible factor driving PVOD. Combination of genetic testing and computed tomography artery facilitated us to distinguish PVOD from PAH. Her disease symptoms advanced aggressively so that she died even before the lung transplantation, which was less than 6 months from the onset of disease symptoms.

**Conclusion:**

This case report highlights that novel *EIF2AK4* mutation at [c.4833_4836dup (p.Q1613Kfs*10)] would predict an aggressive phenotype of PVOD. Hence, we conclude that a genetic test identifying *EIF2AK4* mutation would serve as a tool for the early diagnosis of PVOD, circumventing lung biopsy.

## Background

Pulmonary veno-occlusive disease (PVOD) is a fatal lung disease characterized by diffused occlusion of post-capillary pulmonary venules, intimal thickening and smooth muscle hypertrophy [1]. Clinically, PVOD shares disease phenotype with other forms of pulmonary arterial hypertension (PAH), such as idiopathic PAH [2] or chronic thromboembolic pulmonary hypertension [3]. Vasodilating therapeutics may cause life threatening pulmonary edema in PVOD [4], which necessitates differentiating it from other PAH types.

Eukaryotic translation initiation factor 2 alpha kinase 4 (EIF2AK4), which encodes general control nonderepressible 2 (GCN2) protein has been recently identified as a susceptible factor driving the etiology of PVOD [5, 6]. Recently, a French group investigated the clinical and lung histology data for 24 PVOD patients (12 EIF2AK4 mutation carriers, 12 non-carriers), which reported that pulmonary artery remodeling and decreased GCN2 expression are the common denominators in all cases of PVOD, while the carriers of EIF2AK4 mutation presented a severe intimal fibrosis, stronger muscular hyperplasia of interlobular septal veins, less severe medial hypertrophy and decreased GCN2 expression than the non-carriers [7].

In 2014, Eyries et al sequenced the whole-exome identifying 22 different *EIF2AK4* recessive mutations in 13 families; also identified that 25% of sporadic PVOD patients were carrying biallelic *EIF2AK4* mutation [6]. Later, Tenorio et al reported a founder *EIF2AK4* mutation at c.3344C > T(p.P1115L) in the Iberian Gypsies ethnic group, which caused an early onset of PVOD and even a very low survival rate (1.1 years) after lung transplantation [8]. However, Montani et al described a natural history of more than 8 years in a PVOD patient carrying biallelic *EIF2AK4* mutation at c.[354_355del];[1554–4C.A] [9]. Similarly, another biallelic *EIF2AK4* mutation was reported [c.1392delT(p.Arg465fs)], where the PVOD patient demonstrated compliance with PAH-targeted drugs [10]. These controversies suggest that PVOD is heterogenous displaying variance in disease traits, clinical manifestation, genomics and treatment response [10].

Herein, we present the clinical history of a PVOD patient, who carried a novel and an aggressive version of *EIF2AK4* mutation. We demonstrate that genetic testing distinguishes PVOD from other PAH types.

## Case presentation

A 14-year-old female patient reported shortness of breath, dizziness, and fatigue after minimal physical activity. Based on the combination of echocardiography (ECHO), cardiac magnetic resonance imaging and chest computed tomography, she was diagnosed PAH at a local hospital. Since the disease etiology was unestablished, an immediate pharmacological intervention was not initiated; however, she was supported with oxygen and other symptomatic treatment.

After 4 months, when admitted at our hospital, her blood pressure was 124/84 mmHg, a New York Heart Association functional class III. She was a non-smoker and teetotaler, never abused addictive drugs nor had other PVOD associated risk factors such as chemotherapy with alkylating agents or exposure to organic solvents [5, 7]. And, she had no other physical or psychological illness. Her uncle died of unknown reason at early age, and rest of the family had no history of lung or heart diseases. Her physical examination revealed an accentuated pulmonary component of the second heart sound, tricuspid systolic murmur, and increased breath sound. Her arterial blood gas test indicated 60 and 29 mmHg of PaO_2_ and PaCO_2_, respectively. The concentration of NT-proBNP was 437 ng/mL with normal renal function. The results of coagulation function, thyroid function, HIV, tuberculosis test, rheumatic factor, antinuclear antibody, antiphospholipid antibody, and anti-vasculitis antibody were within the normal range.

**Fig. 1:**
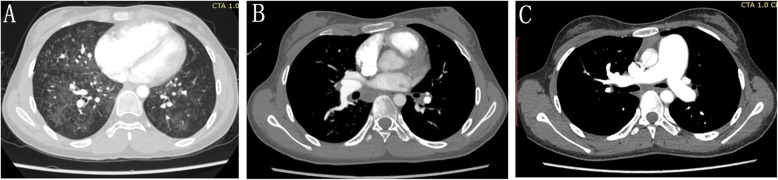
Representative chest CTA image obtained in a *EIF2AK4* mutation PVOD patient. Chest CTA axial section revealed ground-glass opacities and interlobular septal thickening (**a**), enlarged mediastinal lymph nodes (**b**), and a dilated pulmonary main trunk (**c**)

Her ECHO results estimated an elevated right ventricular systolic pressure (80 mmHg), thickened right ventricular anterior wall, dilated right ventricle and right atria, and a normal left ventricle. Chest computed tomography artery (CTA) reported the presence of triad features [5] (subpleural thickened septal lines, ground-glass nodules/opacities, mediastinal lymphadenopathy) and a dilated pulmonary main trunk (Fig. 1), while the right heart catheterization at rest suggested a PAH phenotype (Table 1). Because of her weak physical stature, a lung biopsy was not performed.

**Table 1:** Right heart catheterization results of the patient

	Resistance (wood)	SBP (mmHg)	DBP (mmHg)	MBP (mmHg)	SaO_2_ (mmHg)
Superior vena cava pressure		4	0	2	65.5
Right atria pressure		5	0	2	79
Right ventricular pressure		61	4	27	75.7
Pulmonary artery pressure		62	29	46	75.1
Pulmonary capillary wedge pressure		9	0	4	
Pulmonary vascular	11				
Whole lung	12.1				

*DBP* diastolic blood pressure, *MBP* mean blood pressure, *SBP* systolic blood pressure

In China, congenital heart disease (CHD) predominantly causes pediatric PAH and patent ductus arteriosus (PDA) associated PAH is higher in CHD-PAH [11]. When a PDA-PAH patient develops Eisenmenger syndrome, the elevated pulmonary pressures reverse the shunt from right-to-left making the ECHO assessment difficult [12]. However, our patient had no history of activity restriction or signs of differential cyanosis and clubbing in the upper and lower limbs; therefore, PDA-PAH could be ruled out.

Connective tissue disease (CTD) is the third PAH predisposal factor in China, especially in the young females [11]. Among CTD-PAH, systemic scleroderma (SSc) is prevalent in western countries, while systemic lupus erythematosus (SLE) in Asia [11]. Further, the CTA triad features could be observed in SSc-related PAH [13]. However, our patient showed no signs of SSc- or SLE-PAH or no evidence of respective lab tests (rheumatic factor, antinuclear antibody, antiphospholipid antibody); hence, CHD-PAH was also ruled out.

Interestingly, at least 10% of idiopathic PAH patients fulfill the criteria for PVOD [14]. Mutations in EIF2AK4 gene drive the etiology of hereditary PVOD and also account for 10–25% of sporadic phenotype [4]. Although the PVOD specific bi-basal crackles and digital clubbing was absent, she presented a distinct CTA triad features. Since these radiological changes were insufficient to differentiate PVOD from other PAH types, a genetic testing by whole exome sequencing was performed, which identified a novel *EIF2AK4* mutation at c.4833_4836dup(p.Q1613Kfs*10) (Table 2). This mutation led to a frame shift and the subsequent introduction of a premature stop codon at exon 38, disrupting the expression of this protein.

**Table 2:** Genetic information of the patient from whole exosome sequencing

Gene/transcript	Exosome number	variant^a^	Mutation type	Pathogenicity classification	Genetic mode	Disease/phenotype	RS number
EIF2AK4 NM_001013703.3	38	c4833_4836dup p.Q1613Kfs*10	Het	Likely pathogenic	AR	PVOD	Rs768394773

*AR* autosomal recessive, *PVOD* pulmonary veno-occlusive disease

^a^Variant name followed Human Genome Variation Society nomenclature (http://varnomen.hgvs.org/)

Lung transplantation is the only curative therapy for PVOD [5]. Although she was immediately referred for lung transplantation, she died, before it was scheduled counting the total illness duration to 6 months since first symptom. The timeline of disease process for this patient was showed in the supplementary table.

## Discussion and conclusions

The diagnosis of PVOD is based on the combination of clinical suspicion, physical examination and radiological findings [5, 14, 15]. Our patient presented fatigue and shortness of breath, which are the commonly shared clinical manifestation between PVOD and PAH. Her physical examination didn’t show PVOD-specific features such as bi-basal crackles and digital clubbing [15]. However, her CTA-based triad features indicated an highest possibility of PVOD, but other PAH types like CTD-PAH also demonstrate the triad features [16]. These factors intrigued and sensitized us that a lung biopsy was required to confirm PVOD. Since, our patient was too weak, a surgical biopsy was not performed, hence an additional genetic test identifying *EIF2AK4* mutation could have differentiated atypical PVOD from PAH and arrived the final diagnosis of PVOD, much earlier [4].

Encoded by EIF2AK4, GCN2 is a serine-threonine kinase responsible for the phosphorylation of α–sub-unit of eukaryotic translation initiation factor [6]. GCN2 has been recently shown to inhibit the inflammatory response and systemic autoimmunity triggered by increased cell apoptosis [17]. It is worth to mention that inflammation and autoimmunity contribute to the pathogenesis of PAH [5] and thus, the observed loss of GCN2 might play a prominent role in the pathobiology of PVOD. The *EIF2AK4* mutation in our patient (c.4833_4836dup(p.Q1613Kfs*10) led to the shift of glutamine to lysine at 1613 and the loss of exon 38, presumably a frame shift mutation and premature stop codon. Christina et al reported a splice site *EIF2AK4* mutation at (c.4892 + 1G > T), which also resulted in the loss of the exon 38 [18]. The 31st–39th exons of EIF2AK4 lay in the ribosomal binding domain between the amino acids 1396 and 1643 [19]. Thus premature stop codon caused disrupted protein will at least moderately affect the protein-ribosome binding, driving the PVOD in EIF2AK4 mutation.

Phenotype characterization study demonstrated that carriers of EIF2AK4 mutation usually display an absence of PVOD risk factors, and are susceptible to disease at younger age (median: 26-years vs. 60-years) compared to carriers of non-EIF2AK4 mutation [2]. Similar to previous findings, our patient demonstrated the absence of risk factors such as chemotherapy with alkylating agents or exposure to organic solvents [7] and also was diagnosed at an early age (14 years). Further, PVOD represents poor prognosis such that the current mean time for diagnosis to death and first symptom to death are 14.3 ± 19.3 and 24.7 ± 24.8 months [15]. Likewise, our patient underwent an aggressive disease phenotype in a shorter timeline that her diagnosis to death and first symptom to death were 4 and 6 months, respectively. Overall, combination of CTA triad and identification of novel EIF2AK4 mutation facilitated us to diagnose an aggressive PVOD phenotype without biopsy.

Whole exome sequencing is one of the most prominent next generation sequencing technology, which is widely used in understanding the genetic etiology of a disease; provides an advantage that all genes driving the disease could be investigated in a robust, cheaper and high throughput fashion [20]. With the advancement of technology, the cost of data analysis and interpretation associated with whole exome sequencing has dropped as low as USD 500 per sample; the genetic testing application has rapidly evolved and genetic testing/counseling has been increasing available in many PAH centers [21, 22]. Further, the genetic testing could result in early clinical diagnosis and therapeutic intervention and also curtail down expensive and invasive diagnostic tests. PVOD account for at least 10% of histological form of cases initially considered to be idiopathic PAH [14], which underscore the need of genetic counselling and testing for all suspected patients [21]. Moreover, pre-symptomatic genetic screening of family members of PVOD patients led to careful and regular clinical follow-up of asymptomatic mutation carriers, facilitating early diagnosis [21]. Given those considerations and the severity of misdiagnosis, it would be prudent to provide genetic testing for the suspected PVOD or idiopathic PAH patients. This implementation would allow an early diagnosis and a better genotype–phenotype correlation [8]. Our report lacks lung biopsy, which is a limitation.

To surmise, PVOD remains challenging at both diagnostic and therapeutic interventions. Because of poor prognosis, aggressive disease symptoms and therapeutics related concerns, it is crucial to identify the PVOD cases in a timely fashion. Genetic testing for EIF2AK4 mutation would facilitate the diagnosis and prognosis of PVOD circumventing the requirement of a lung biopsy. Further, the novel *EIF2AK4* mutation at [c.4833_4836dup (p.Q1613Kfs*10)] predicted an aggressive phenotype, which would serve as a biomarker in assessing the rapid progression of PVOD. Future study warrants the examination of EIF2AK4 mutation against the development of PVOD.

## Supplementary information

**Additional file 1: Supplementary table.** Timeline of disease process for this PVOD patient.

## Data Availability

Data sharing is not applicable to this article as no datasets were generated or analyzed during the current study.

## Abbreviations

CHD: Congenital heart disease
CTA: Computed tomography artery
CTD: Connective tissue disease
ECHO: Echocardiography
EIF2AK4: Eukaryotic translation initiation factor 2 a kinase 4
GCN2: General control nonderepressible 2
PAH: Pulmonary arterial hypertension
PDA: Patent ductus arteriosus
PVOD: Pulmonary veno-occlusive disease
SLE: Systemic lupus erythematosus
SSc: Systemic scleroderma
