# Supplementary material for: Rapid disease progress in a PVOD patient carrying a novel EIF2AK4 mutation: a case report
Source: BMC Pulm Med. 2020 Jul 6;20:186. doi: 10.1186/s12890-020-01186-8 (PMC7336641; doi:10.1186/s12890-020-01186-8)
Supplement: Supplementary file 1 — Additional file 1: Supplementary table. Timeline of disease process for this PVOD patient. [file 12890_2020_1186_MOESM1_ESM.docx]

**Supplementary table: Timeline of disease process for this PVOD patient**

| DATA | Relevant Past Medical History and Interventions | | |
| --- | --- | --- | --- |
| **In the past** | She was a non-smoker and teetotaler, never abused addictive drugs nor had other PVOD associated risk factors. And she had no other physical or psychological illness. Her uncle died of unknown reason at early age, and rest of the family had no history of lung or heart diseases. | | |
| DATA | **Summaries from Initial and Follow - up Visits** | **Diagnostic Testing**  **(including dates )** | **Interventions** |
| **20190126-20190130** | This patient was admitted to the local hospital for progressive shortness of breath, dizziness, and fatigue after minimal amount of activity. Based on the combination of echocardiography, cardiac magnetic resonance imaging and chest computed tomography, she was diagnosed with PAH at a local hospital. | 20190126-20190128:  Laboratory test;  20190128: Echocardiography;  20190129: Cardiac MRI;  20190129: Chest CT | Oxygen inhalation and other supportive symptomatic treatment were given. |
| **20190403-20190416** | Four months after her first symptoms, she was admitted to our hospital with the complaint of progressive shortness of breath. Based on her physical examination and related image studies, the initiated diagnosis of PDA-PAH, CTD-PAH and PVOD was considered. Combined with CTA findings, her genetic testing identify a novel *EIF2AK4* mutation which facilitated the final diagnosis of PVOD. | 20190403-20190410:Laboratory test  20190404: Chest x-ray;  20190405:Echocardiography;  20190405:Chest CTA;  20190409: RHC;  20190414: Genetic testing; | Oxygen inhalation;  Low salt, low fat diet;  Electrocardiogram monitoring;  Life symptoms, blood gas level, and blood electrolytes Monitoring;  She was immediately referred to another facility for lung transplantation when the diagnosis of PVOD was established. |
| **20190706** | Follow - up visits: Three months after her discharge, we followed this patient by telephone call; she died of sudden death in June, 2019. | | |
|  | Final outcome for this episode of care: This patient died of sudden death before the initiation of lung transplantation with the total illness duration of 6 months since the beginning of symptoms | | |

CT: computed tomography; CTA: computed tomography artery; CTD: Connective tissue disease; PDA: patent ductus arteriosus; PVOD: pulmonary veno-occlusive disease; MRI: magnetic resonance imaging: RHC: right heart catheterization
